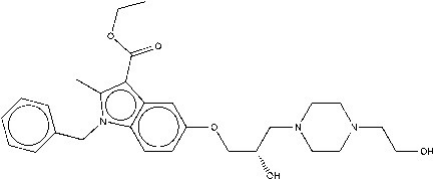# Systems biology approaches based discovery of a small molecule inhibitor targeting both c-Met/PARP-1 and inducing cell death in breast cancer

**DOI:** 10.7150/jca.40758

**Published:** 2020-02-19

**Authors:** Tian Yu, Lijia Cheng, Xueling Yan, Hang Xiong, Jie Chen, Gang He, Hui Zhou, Hongbo Dong, Guangya Xu, Yong Tang, Zheng Shi

**Affiliations:** 1 School of Medicine & Sichuan Industrial Institute of Antibiotics & Department of Respiratory and Critical Care Medicine, Affiliated Hospital/ Clinical College of Chengdu University, Chengdu University, Chengdu 610015, China; 2 School of Acupuncture and Tuina, Chengdu University of Traditional Chinese Medicine, Chengdu 610075, China; 3 Central Laboratory of Clinical Medicine, Sichuan Academy of Medical Science and Sichuan Provincial People's Hospital, Chengdu, 610000, China

**Keywords:** breast cancer, c-Met, PARP-1, systems biology, apoptosis, drug discovery

## Abstract

Breast cancer is the second most common types of cancer worldwide. Molecular strategies have developed rapidly; however, novel treatments strategies with high efficacy and lower toxicity are still urgently demanded. Notably, biological networks estimated from microarray data and functional activity network analysis could be utilized to identify and validate potential targets. In this study, two microarray data (GSE13477, GSE31192) were firstly selected, and analyzed by multi-functional activity network analysis to generate the core protein-protein-interaction (PPI) network. Several potential targets were subsequently identified and c-Met and poly (ADP-ribose) polymerase-1 (PARP-1) were manually chosen as the key targets in breast cancer. Furthermore, virtual screening and molecular dynamics (MD) simulations were utilized to recognize novel c-Met/PARP-1 inhibitors in Specs products database. Three small molecules, namely, ZINC19909930, ZINC20032678 and ZINC13562414 were selected. Additionally, these compounds were synthesized, and two breast cancer cell lines, MDA-MB-231 and MCF-7 cells were used to validate our bioinformatic findings *in vitro*. MTT assay and Hoechst staining showed that ZINC20032678 significantly induced breast cancer cell death, which was mediated through apoptosis by flow cytometry. Furthermore, ZINC20032678 was shown to target the active sites of the both targets and recruitment of downstream apoptotic signaling pathways, eventually inducing breast cancer cell apoptosis. Collectively, our findings not only offer systems biology approaches based drug target identification, but also provide the new clues for developing novel inhibitors for future breast cancer research.

## Introduction

Breast cancer is one of the most leading causes of cancer death among women. It is reported that the overall pathogenic incidence rate in women remains generally stable, whereas the breast cancer incidence has slight increased from 2004 to 2013 1.Currently, there are four main molecular subtypes of breast cancer, referring as luminal, HER2, normal-like and basal. Of note, targeted therapies have been raised great attention in science community. It can target specific targets expressed in/on the surface of cancer cells which are involved in carcinogenesis and/or tumor growth 2. For example, tamoxifen for treatment of ER positive tumors and trastuzumab in the treatment of HER2 positive tumors in breast cancer have been extensively studied 3. However, it is worth knowing that most basal-like breast cancers do not express ER, PR and HER2, the two subgroups are not mutually exclusive. And, 80% of basal-like breast cancers are triple-negative and 80% of triple-negative breast cancers exert a basal-type phenotype 4. Therefore, fundamental breakthroughs, particularity new therapeutic targets and new drugs are most urgent needed in breast cancer research.

It is known to all that the entire DNA microarrays has been used in cancer drug development and facilitate clinical applications for years 5. Since measurement of the sequence and expression of potential targets is greatly facilitated by microarray technology, it is of great importance to generate new clues to gene function which can help to identify appropriate targets for therapeutic intervention. Hitherto, a great deal of drug targets discovery and validation have been well described by microarray analysis 6, 7. Additionally, DAVID online database, an integrated biological knowledge base and analytic tool, can extract biological meaning from large gene/protein lists 8. Moreover, STRING database includes known and predicted PPIs which stem from various databases 9. Combining the microarray data and functional activity network analysis of differentially expressed genes by using DAVID (including KEGG pathway and GO analysis) and STRING were accelerate the drug target development 10. To our knowledge, a number of hub proteins/targets were successfully identified by using abovementioned approaches 11, 12.

In this study, two microarray data (GSE13477, GSE31192) were firstly analyzed to obtain consensus results of differently expressed genes. Then, the up- regulated genes were selected to identify the candidate genes by using GO, KEGG pathway and STRING analysis. According to the network results and consulting numerous references, c-Met and PARP-1 were manually selected as the key targets in breast cancer. Subsequently, molecular docking and MD simulations were performed to recognize valuable agents to target both c-Met/PARP-1. Then, three small molecules, namely ZINC13562414, ZINC20032678 and ZINC19909930 were identified as potential inhibitors against both c-Met and PARP-1. We further synthesized three compounds, and cultured MDA-MB-231 and MCF-7 cells and treated with these compounds. Eventually, the compound ZINC20032678 effectively induced breast cancer cell apoptosis through inhibition of both c-Met and PARP-1 kinases activities. In general, our findings would lead to comprehensive mechanistic insights into identification of more ideal targets as well as more novel potential drugs in future cancer drug discovery.

## Material and methods

### Hub protein identification

**Figure 1:**
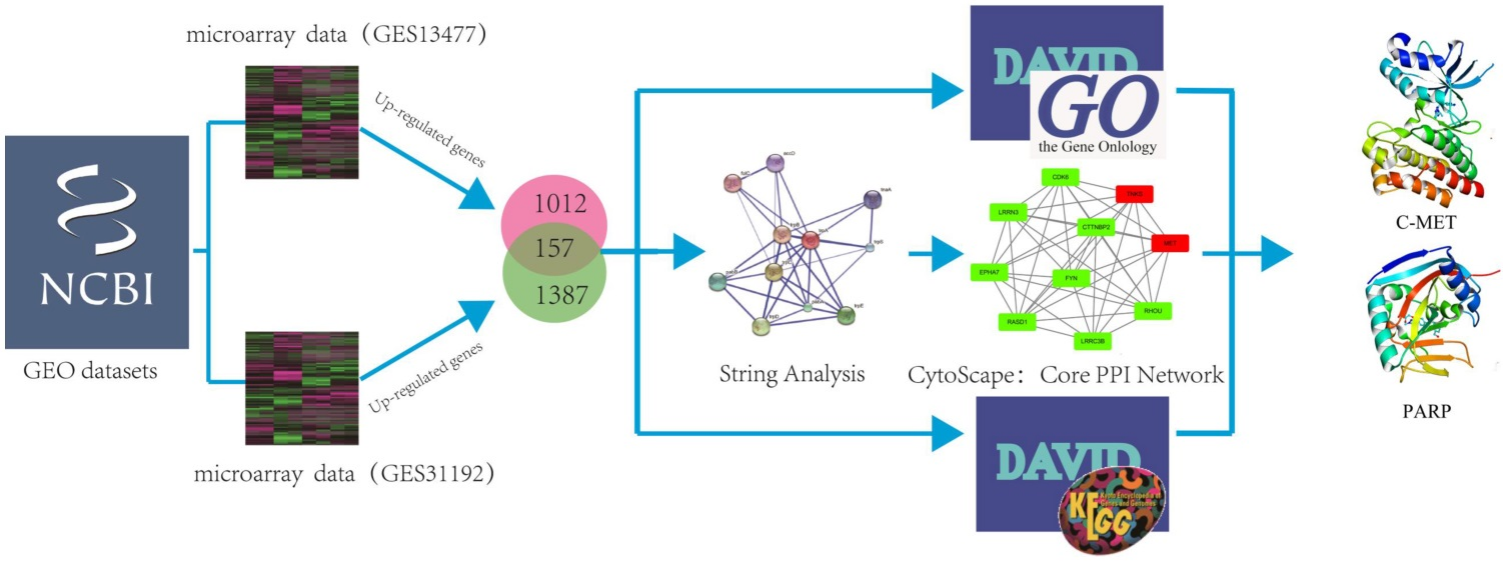
The schematic model of data mining and virtual screening process. We systematically combined two microarray datasets and analyzed these data by using GO and pathway enrichment analysis to predict hub proteins in breast cancer.

In order to investigate if genes were co-expressed or not, two microarray data (GSE13477; GSE31192) under the determiners “Breast cancer” AND “*Homo sapiens*” from the GEO Data Sets were selected, and analyzed by using GEO2R tool (https://www.ncbi.nlm.nih.gov/geo/geo2r/). Two microarray data by using different text conditions were selected. The aim of two chips is to identify differential genes between normal tissues and cancer (cells), while excluding the error of individual chip experiments through cell chips and tissue chips. GO analysis could provide comprehensive information on gene function of individual products through ontology. An Expression Analysis Systematic Explorer (EASE) score < 0.1 was adopted to refine the GO terms set in major clusters through Database for Annotation, Visualization and Integrated Discovery Bioinformatics Resources version 6.8 (DAVID:https://david.ncifcrf.gov/). A protein interaction network of each gene and all protein interaction networks of up-regulated genes and down-regulated genes were constructed through STRING. Subsequently, the selected genes were carried up by using DAVID to get the KEGG pathway and Gene Ontology results, and using STRING to get the core PPI network, respectively. We further imported the results into cytoscape to strip out the core PPI network. Herein, the identification of the hub protein schematic model was shown in Figure 1.

### Molecular docking

The initial 3D X-ray crystal structures of c-Met (3ZCL) and PARP-1 (3L3M) were downloaded from the RCSB Protein Data Bank (PDB) (http://www.pdb.org/pdb/home/home.do) 13. 32791 candidate compounds that were commercially available for screening were collected from specs subsets (http://zinc.docking.org/catalogs/specsnp) 14. The c-Met/PARP-1 and their ligands were prepared by USCF chimera (Version1.8) by elimination of solvent molecules as well as insertion of hydrogen atoms and standard charges 15. UCSF DOCK (version 6.5) was used to identify compound libraries against a target receptor to predict potential drugs 16, 17. To ensure the accuracy of docking, the specified parameters docking were confirmed by redocking the co-crystallized ligand back into the binding site of the receptor. Root mean square deviation (RMSD) serves as a reference value for judging docking performance 18, 19. The top 100 ranked small molecular natural products were rescored by amber score function 20, 21. Generally speaking, the lower score of ligands indicates the higher binding affinity to receptor.

### MD simulations

GROMACS package (Version 4.5) 22 was adopted to perform the MD simulations. Amber ff99SB force field and TIP3P water mode were explored to convert the protein structure into the topology 23. Additionally, the ligands were charged by AM1-BCC via antechamber program. Then, parmchk program was utilized to check and revise the missing force field parameters as well as converting the parameters and topologies for ligands with ACPYPE tools 24. Simulations were carried out under periodic boundary conditions (PBC) with a dodecahedron periodic box setting the minimal distance of 1.0 nm between the protein and edge of the box, which was solvated by adding simple point charge (SPC) water molecules 25. Moreover, Na^+^ and Cl^-^ were added to mimic a physiological NaCl concentration of 0.15 M to neutralize the solvated system. Subsequently, we employed the steepest descent minimization algorithm for all atoms to minimize the energy of system 26. Afterwards, 100 ps NVT (constant number of particles, volume, and temperature) and 100 ps NPT (constant number of particles, pressure, and temperature) equilibrations were performed for each system with position restraints for receptor and potent small molecules. In the end, 25 ns MD simulations with a time step of 2 fs at constant pressure (1 atm) and temperature (300 K) were performed. During the MD simulation process, all bond lengths were constrained by LINCS algorithm and Particle Mesh Ewald (PME) method was adopted to calculate the long-range electrostatic interactions 27-29. The trajectories of c-Met/PARP-1 bounded with different small molecules were computed by backbone root mean square deviation (RMSD, g_rms) of GROMACS utilities.

### Chemical synthesis of candidate compounds

**Figure 2:**
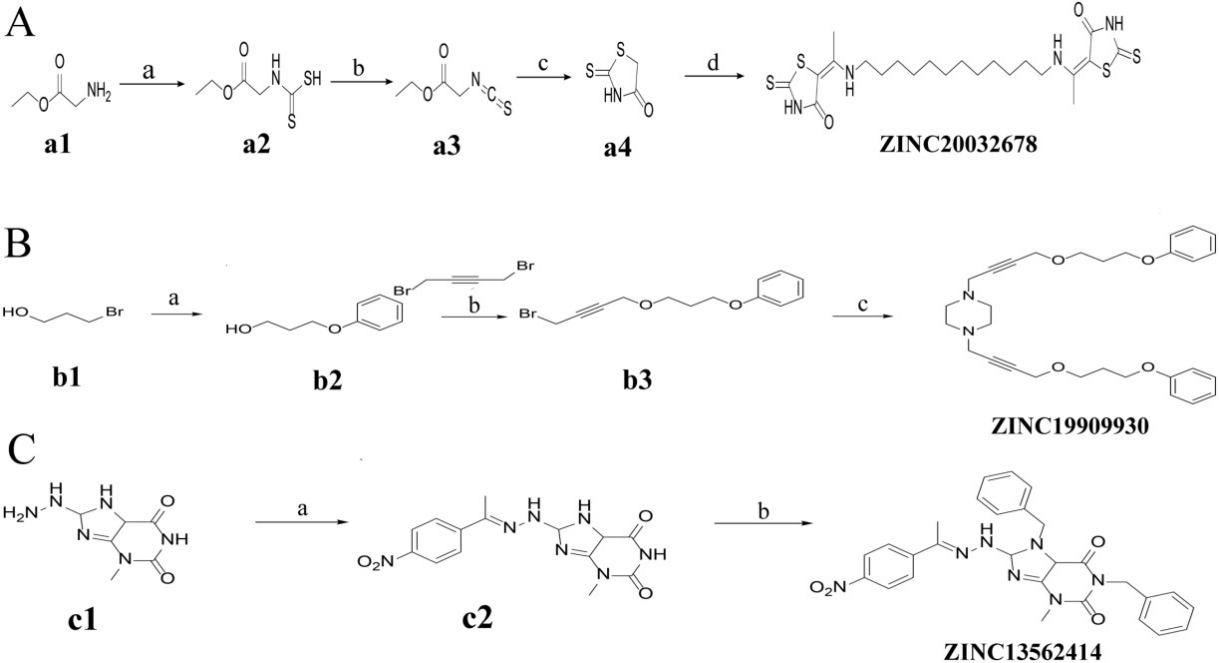
Synthetic route of three compounds. (A) Synthetic Approach to ZINC20032678. Reagent and conditions: (a) CS_2_, water, ambient temperature, 48 h; (b) HgCl_2_, methylbenzene, 5h; (c) DMF, 120 ºC, 6h; (d) CH_3_CHO, NH_2_(CH_2_)_12_NH_2_, 6h. (B) Synthetic Approach to ZINC19909930. Reagent and conditions: (a) 18-crown-6-ether, K_2_CO_3_, acetone, 12h; (b) NaOH, 1.4-dibromobut-2-yne, N-Benzyl-N-triethylammonium chloride, benzene, 70 ºC, 15h; (c) piperazine, NaOH, acetone. (C) Synthetic Approach to ZINC13562414. Reagent and conditions: (a) 1-(4-nitrophenyl)etana-1-one, KOH/K_2_CO_3_, Water, 0.5h; (b) (bromomethyl)benzene, NaOH, DMF, 4h.

Synthesis of ZINC20032678 requires two key intermediates **a4** and dodecane-1, 12-diamine, which were synthesized using the route described in Figure 2A (ZINC20032678). Dodecane-1, 12-diamine was commercial availability. To prepare the intermediates **a4**, compound a1 was treated with CS_2_ and heated in the water to furnish the corresponding carbamodithioic acid **a2**. Compound **a2** was oxidized by HgCl_2_ to afford ethyl, 2-isothiocyanatoacetate **a3**. Finally intermediate **a4** could be obtained from **a3** through cyclization in DMF at 120 C.

For ZINC19909930, our synthetic strategy started from commercially available 3-bromopropan-1-ol **b1**.Compound **b1** was treated with phenol and then 1, 4-dibromobut-2-yne to get key Intermediate **b3**. Compound **b3** was reacted with piperazine to get ZINC19909930 in good yield (Figure 2B).

As for compound ZINC13562414, our synthetic strategy started from compound **c1**. Compound **c1** was reacted with 1-(4-nitrophenyl) ethan-1-one to get compound **c2**. The amino hydrogens of compound **c2** were replaced by benzyl groups to get ZINC13562414 (Figure 2C).

### Cell culture

The MDA-MB-231 and MCF-7 cells were purchased from American Type Culture Collection (ATCC, Manassas, VA, USA). The cells were cultured in Dulbecco's Modified Eagle Medium (DMEM) with 10% fetal bovine serum (FBS) and 1% penicillin/ streptomycin, and maintained in a 5% CO_2_/37℃ incubator, respectively. Cells were using with 0.25% trypsin, DMEM, FBS and antibiotics were purchased from GIBCO (Carlsbad, CA, USA).

### (4,5-dimethyl-2-thiazolyl)-2,5-diphenyltetrazolium bromide (MTT) assay

5✕10^3^ MDA-MB-231 and MCF-7 cells were seeded in 96-well plate, respectively. Eighteen hours post-seeding, cells were treated with different concentrations of ZINC20032678 as followings: 0.1 μmol/L, 1 μmol/L and 10 μmol/L ZINC20032678. Cisplatin was used as positive control, and untreated cells were set as blank control. Each group was set three wells. Forty-eight hours post-drug treatments, the viable cells were stained by adding 20 μl of 5 mg/ml MTT solution per 100 μl of growth medium. After incubating for 4 h at 37℃, the media were removed and 150 μl DMSO was added to dissolve the formazan. The absorbance of each well was measured by microplate reader and viable cells are presented as a percent of the control.

### Hoechst staining

1✕10^5^ MDA-MB-231 and MCF-7 cells were seeded in 6-well plate, respectively. The cells were treated with different concentrations of small molecule ZINC20032678 as followings: 0.1 μmol/L, 1 μmol/L and 10 μmol/L ZINC20032678 after 18 h. Cisplatin was used as positive control and untreated cells were set as blank control. Each group was set three wells. Forty-eight hours later, cells were stained with Hoechst kit from Beyotime (Haimen, Jiangsu, China). Cell counting was carried out using the software ImageJ from National Institutes of Health, which is available at http://rsbweb.nih.gov. The corresponding cell death rates were calculated according to Hoechst staining.

### Annexin V-FITC/PI stained fluorescence-activated cell sorter (FACS)

To explore whether the cell death was caused by apoptosis, MDA-MB-231 cells was chosen to perform Annexin V-FITC/PI stained FACS. After incubation of cells with 0.1, 1 and 10 μmol/L ZINC20032678 for 48 h in 6-well plate, the cells were harvested through trypsinization, and washed twice with PBS. The cells were centrifuged at 3000 r/min for 5 min, then the supernatant was discarded and the pellet was resuspended in 1×binding buffer at a density of 1.0×10^5^/ml. The sample solution of 100 μl was transferred to a 5 ml culture tube, and incubated with 5 μl FITC-conjugated annexin V (Abcam, USA) and 5 μl PI (Abcam, USA) for 15 min at room temperature in the dark. 400 μl 1×binding buffer was added to each sample tube, and the samples were analyzed by FACS (Mindray, China).

### Western blot

Proteins were extracted from MDA-MB-231 cells treated with 0, 0.1, 1 and 10 μmol/L ZINC20032678 and 1 μmol/L crizotinib (c-Met inhibitor) or NMS-P118 (PARP-1 inhibitor) at 72 h, respectively, and the untreated cells were set as the blank control. Then we further separated by using a 10% polyacrylamide gel. After transferring the protein on a nitrocellulose membrane, the membrane was blocked with a 5% defatted milk solution and probed with recombinant monoclonal antibody against phosphorylated c-Met (1:2000, Abcam, USA), full-length monoclonal PARP-1 (1:1000, Abcam, USA), cleaved PARP-1 (1:1000, Abcam, USA), cleaved caspase-3 (1:500, Abcam, USA), Bcl2 (1:2000, Abcam, USA), Bax (1:2000, Abcam, USA) and β-actin (1:5000, Abcam, USA), and then probed with a secondary antibody using ALP conjugated anti-human IgG (1:5000, Santa cruz, USA), at last, blots were developed using a ECL plus kit (GE, USA).

### Statistically analyses

Data are expressed as means ± standard deviation 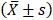 and were analyzed by ANOVA analysis (SPSS 22.0, USA). A *P*<0.05 was considered statistically significant.

## Results

### Identification of hub proteins in breast cancer

**Figure 3:**
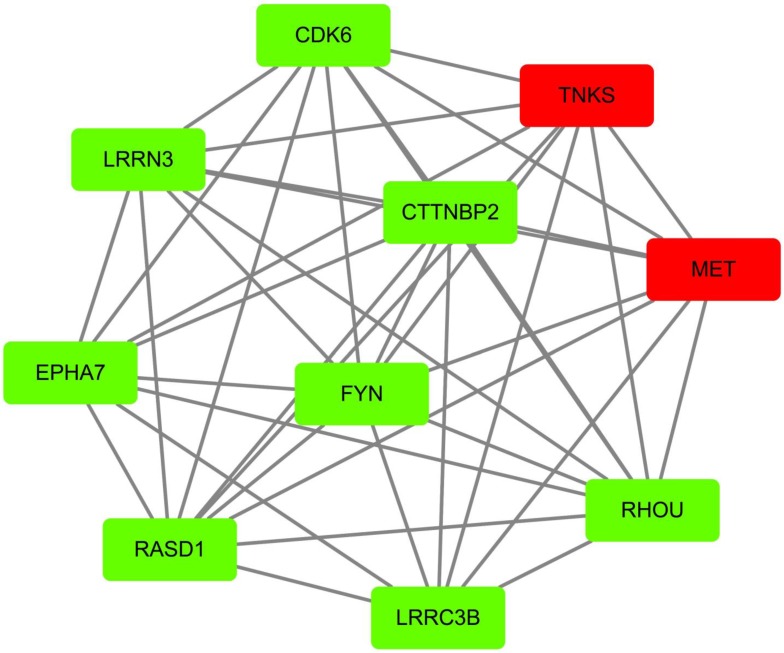
Identification of core PPI network in breast cancer. Co-expressed network of up-regulated differently expressed genes presented by node and edge by the analyzer of cytoscape. The red nodes indicate more protein that could interact with this node, while the green node indicates less protein integrators.

According to GEO datasets, 1169 up-regulated genes and 1544 up-regulated genes were subsequently obtained under the accession number GSE 13477 and GSE 31192, respectively. To avoid false positive results, the consensus results including 157 up-regulated genes were finally selected. These up-regulated genes were analyzed by using DAVID 6.7 to get results of GO analysis and KEGG-pathway analysis, respectively. Genes with* P* value (*P*<0.0054) were chosen from of GO analysis, and genes with minimum *P* value were also selected from KEGG-pathway analysis. At the same time, we analyzed the 157 up-regulated genes by using STRING database to obtain PPI network, then peeling sub-network to obtain core PPI network by using cytoscape software, and genes which had the maximal degree value were eventually chosen. And, GO enrichment analysis and comprehensively integration of results from GO analysis, pathway enrichment and cytoscape software analysis, the core PPI sub-network was established (Figure 3). Hub proteins, such as c-Met, PARP-1, EGFR, IGF1, DASD1 were finally recognized (Table 1). After consulting numbers of references, c-Met and PARP-1 were considered as the key targets in breast cancer.

### Virtual screening for potential c-Met/PARP-1 inhibitors

32791 compounds from specs chemistry database were launched to recognize both c-Met and PARP-1 inhibitors by virtual screening. After the docking procedure, the detailed information of Top 10 ideal results was shown in Table 2. Subsequently, we finally chose 5 small molecules, namely ZINC13562414, ZINC20032678, ZINC19909930, ZINC19909927, and ZINC06444965 for MD simulations to evaluate the dynamic interactions between c-Met/PARP-1 and their potential ligands within the given period of time.

### MD simulations

**Figure 4:**
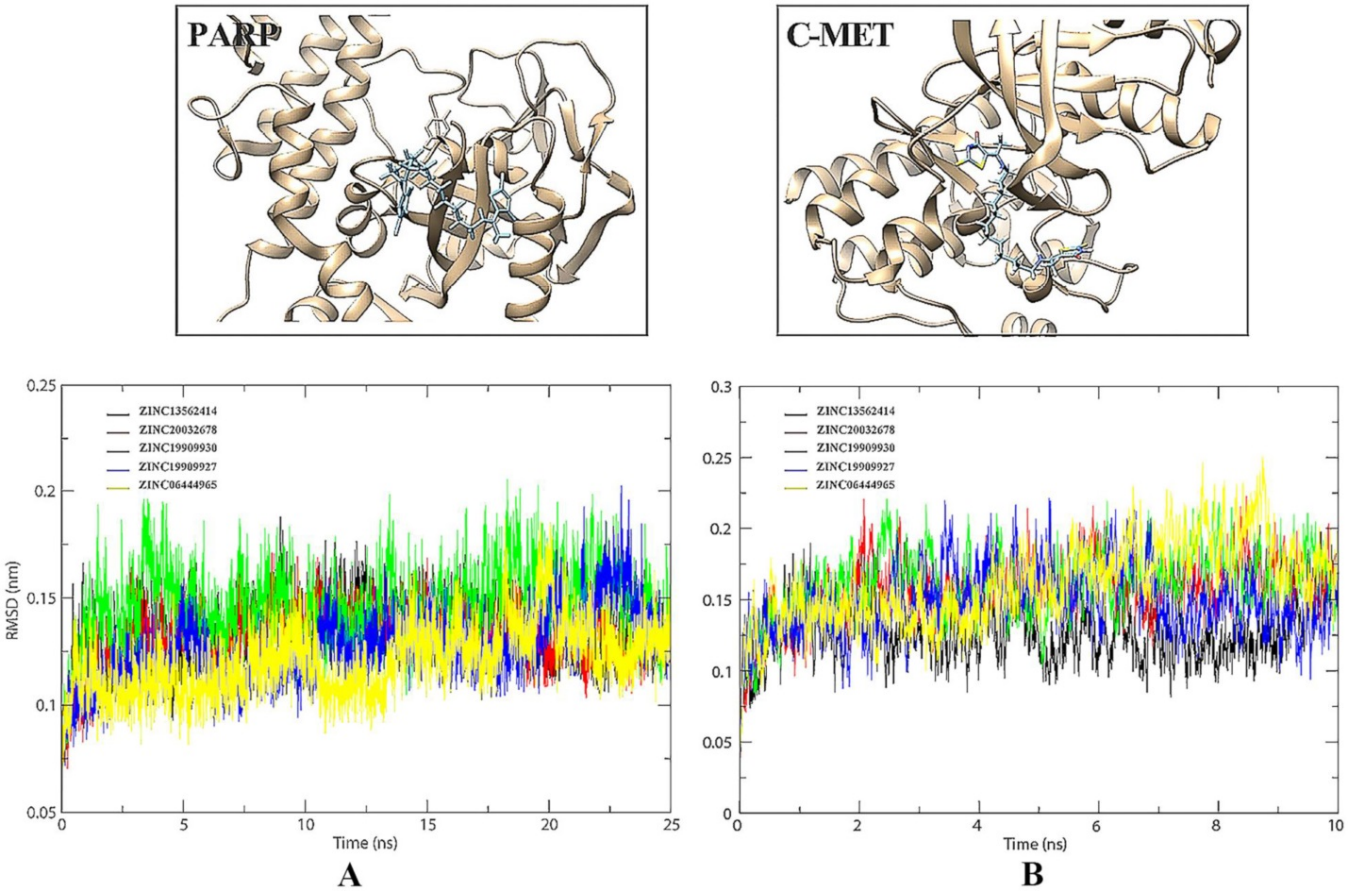
(A) Comparison of the binding site before and after docking of PARP-1 and its initial ligands. (B) Comparison of the binding site before and after docking of c-Met and its initial ligands.

RMSD value is an important indicator of stability of drug-target interaction. Herein, the backbone RMSD values were calculated to assess the stability of the protein-ligand binding models during the MD simulations. We took 25 ns MD simulations to evaluate the dynamic interactions between two targets and their ligands. As was shown in Figure 4A, the 5 selected small molecules had backbone RMSD values ranging from 0.075 to 0.20 nm. Given their starting structures, c-Met-ZINC19909930 and c-Met-ZINC13562414 showed similarly sharp rise during the first 2.5ns. ZINC20032678 reached equilibrium after the initial period of fluctuation with the values of backbone RMSDs around 20 nm. c-Met-ZINC06444965 system had the trend of rise within 5 ns, while the c-Met-ZINC19909927 system exhibited maximum deviation. Compared to other systems, ZINC13562414, ZINC20032678, ZINC19909930 systems were relatively balanced, indicating these three small molecules had stronger affinities, towards c-Met. Similarly, Figure 4B indicated that PARP-1-ZINC19909930, ZINC13562414 and ZINC20032678 systems exerted relatively balance than the other two systems. Therefore, these three molecules were selected for further *in vitro* study.

### Effects of compounds on breast cancer cell viability

**Figure 5:**
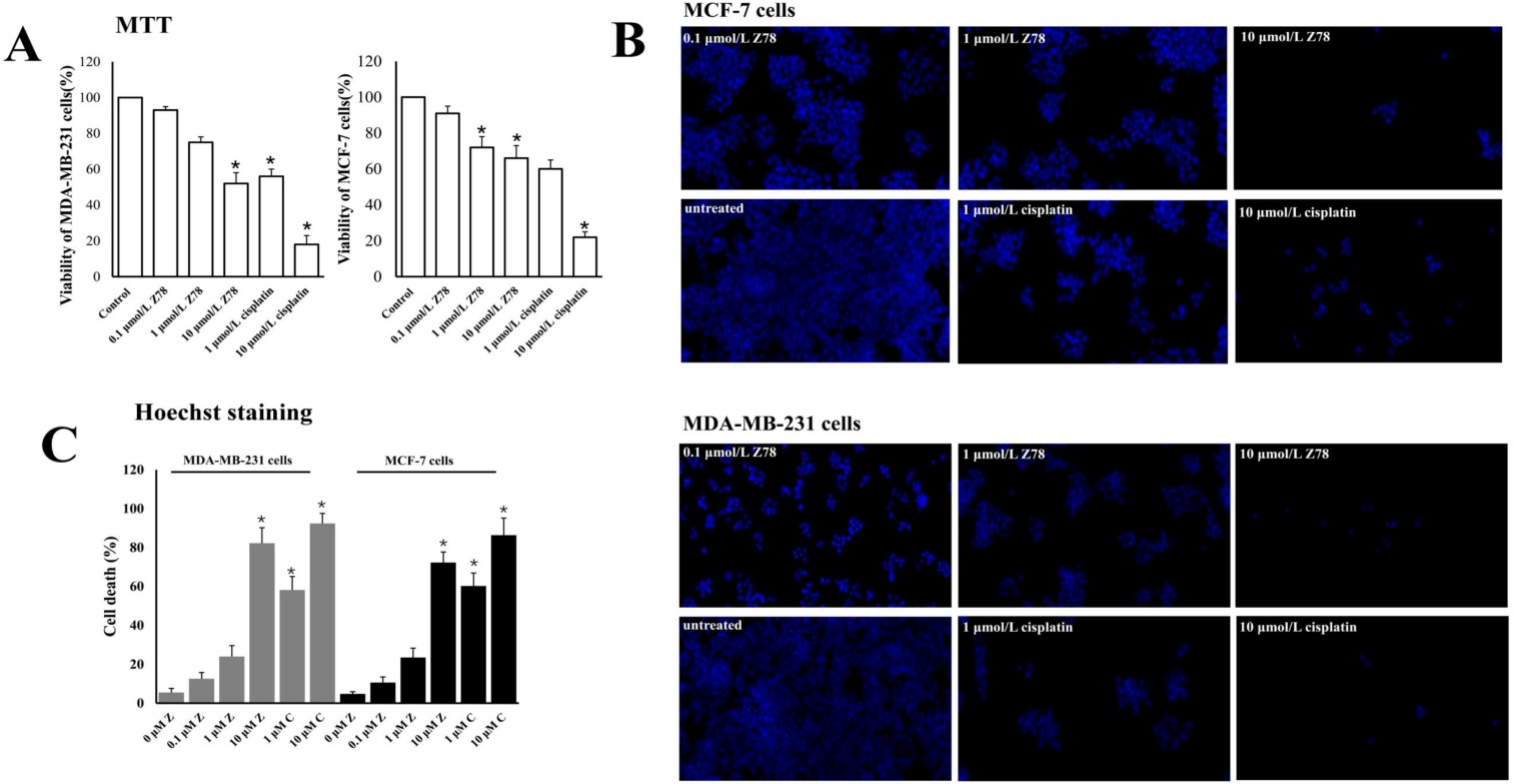
(A) The viability of breast cancer cells, MDA-MB-231 and MCF-7 cells, was detected by MTT assay. The cell growth was significantly inhibited by 10 µmol/L ZINC20032678 at 48 h. (B) The Hoechst staining of MDA-MB-231 and MCF-7 cells treated with ZINC20032678 or cisplatin which induce cell death. Bar: X40. (C) The percentage of cell death according to Hoechst staining at 48 h. *P<0.05. Z78: ZINC20032678.

To determine whether the treatment of three compounds affected the cell survival abilities, we examined the cell viability by MTT assay after drug treatment for 48 h post-drug treatments. Results demonstrated that the cell survival abilities of both MDA-MB-231 and MCF-7 cells were significantly decreased only in ZINC20032678 treated group (*P*<0.05), not in the other two compound-treatment groups. The effects of ZINC20032678 were similar to that of the positive control group (Figure 5A). Interestingly, such effect was also in a dose-dependent manner. These results indicated that the ZINC20032678 could effectively inhibit the breast cancer cell survival.

To further investigate the effects of ZINC20032678 on the cell survival rate, we investigated the percentages of cell death of the two cell lines, upon ZINC20032678 treatment by Hoechst staining. Compared to the untreated group, the percentage of dead cells was significantly increased in group treated with 10 μmol/L ZINC20032678 and cisplatin (Figure 5B&C). And the effect of inhibiting cell activity was better in MDA-MB-231 cells. Therefore, we chose MDA-MB-231 cells to perform the further study.

### Effects of ZINC20032678 on apoptosis of breast cancer cells

**Figure 6:**
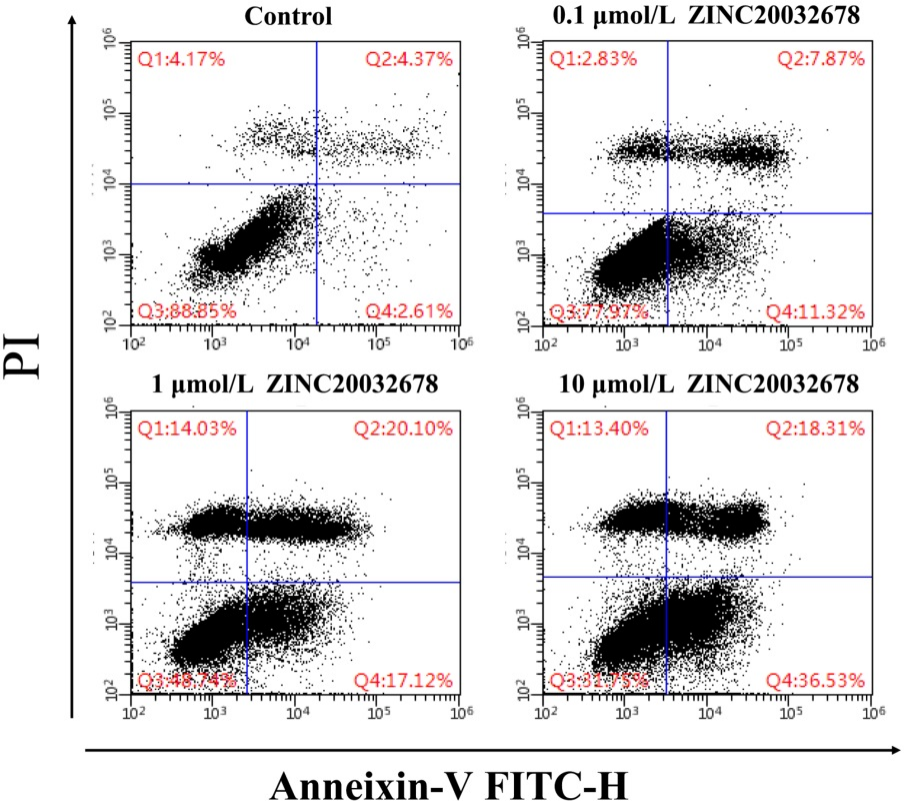
Flow cytometry of Annexin V-FITC as a proxy for small molecular ZINC20032678 induced cellular apoptosis in MDA-MB-231 cells line for 48 h. (A) There weren't obvious apoptosis in breast cancer cell lines in blank control group; (B) The percentage of early apoptosis cells was 11.43% in 0.1 μmol/L ZINC20032678 group; (C) The percentage of early apoptosis cells was 17.12% in 1 μmol/L ZINC20032678 group; (D) The percentage of early apoptosis cells was 36.53% in 10 μmol/L ZINC20032678 group.

To identify the cause of the breast cell death, the Annexin V/PI staining with MDA-MB-231 cells was run by flow cytometry after treated with 0, 0.1, 1 and 10 μmol/L ZINC20032678 for 48 h. From the results we could see that the percentage of early apoptotic cells was 11.32%, 17.12% and 36.53% after treatment of 0.1, 1 and 10 μmol/L ZINC20032678, respectively; while the blank control did not induce obvious cell apoptosis (Figure 6). Therefore, the compound ZINC20032678 could induce breast cancer cell apoptosis.

### Inhibition of c-Met and PARP-1 kinases activities *in vitro*

**Figure 7:**
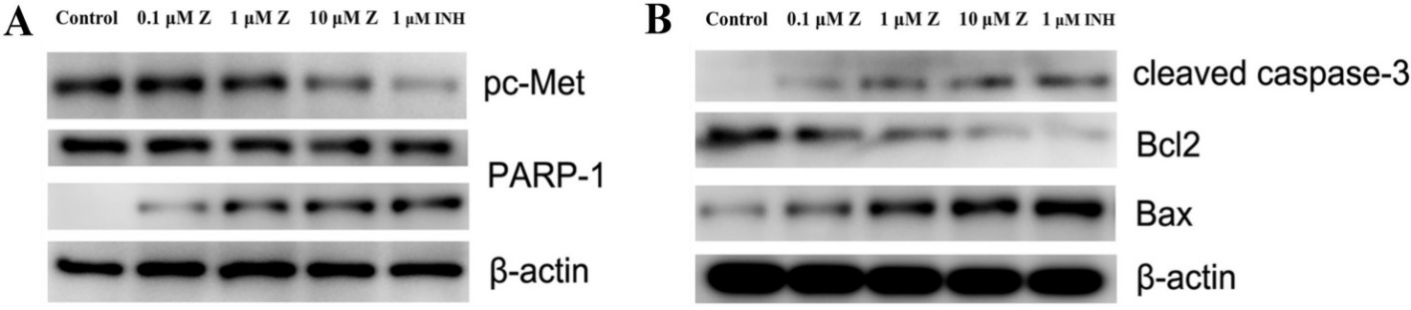
(A) The expression of phosphorylated c-Met, full-length and cleaved PARP-1 secreted by MDA-MB-231 cells at 72 h with western blot. (B) The expression of relative proteins of c-Met/PARP-1 downstream apoptotic signaling pathway, cleaved caspase-3, Bcl2 and Bax were detected by western blot.

ZINC20032678 was identified to target both c-Met/PARP-1 proteins by series of *in silico* approaches. Therefore, the expression of phosphorylated c-Met and PARP-1 were detected by western blot, in which, the inhibitor of c-Met was crizotinib, and the inhibitor of PARP-1 was NMS-P118. In addition to this, there were two forms of PARP-1, full-length PARP-1 and cleaved PARP-1. The results showed that c-Met was significant inhibited by 10 μmol/L ZINC20032678 and its inhibitor; while ZINC20032678 and NMS-P118 disintegrated the full-length RARP-1 into cleaved PARP-1 (Figure 7A). Our results suggested that apoptosis could be induced in breast cancer cells through inhibition of c-Met/PARP-1 kinases activities.

After inhibition of c-Met/PARP-1 kinases activeties, the downstream apoptotic signaling pathways were then activated, the cleaved capase-3 was activated, and the expression of Bcl-2 was inhibited while the expression of Bax was up-regulated treated by ZINC20032678 or the corresponding inhibitors (Figure 7B). Therefore, ZINC20032678 could target both c-Met/PARP-1 sequentially activating the downstream apoptotic signaling pathway, eventually inducing breast cancer cell apoptosis.

## Discussion

Of note, breast cancer is a heterogeneous disease which comprises a variety of pathologies and displays a range of histological characteristics and clinical outcomes. Systems biology approaches offer novel perspectives to reveal the molecular mechanisms of cancer, and thus shedding light on the identification of potential drug targets for future anti-cancer drug discovery 30, 31. Currently, the identification and validation of targeted therapy by using systems biology approaches are the most promising strategies in breast cancer research 32, 33. Previous studies have indicated novel apoptotic kinase targets, namely AMPK and ZIPK, in cervical cancer by using systems biology methods. And, they screened and discovered a novel dual target activator targeting AMPK/ZIPK and inducing apoptosis in cervical cancer. Such small molecule named BL-AD008 is considered as a promising candidate agent in cervical cancer drug development 34. In this study, multiple approaches including microarray analysis, biological characteristics were utilized to screen the hub proteins, and c-Met/PARP-1 were considered as the most effective drug targets in the context of breast cancer.

c-Met is an oncogene which encodes a membrane-bound tyrosine kinase implicated in the formation and/or progression of various types of tumors. It is reported that overexpression of c-Met has been discovered in a number of studies and with poor diagnosis in breast cancer 35. Thus, inhibition of c-Met has become an emerging therapeutic strategy for breast cancer. Bulks of studies have highlighted the effects of c-Met deregulation on carcinogenesis and development of aggressive phenotypes in breast cancer 36. Moreover, previous studies also indicated that combined therapy targeting both c-Met and EGFR may be beneficial for the treatment of breast cancer patients. Moreover, another study suggested c-Met overexpression was detected in 20-30% of breast cancer patients, which might be associated with a worse prognosis, suggesting that inhibition of c-Met might be an attractive oncology therapeutic approach 37. Hitherto, a variety of small molecule kinase inhibitors targeting c-Met have been evaluated in different stages of clinical trials 38. For example, Foretinib, a multi-target kinases inhibitor against both c-Met and VEGFR2, is currently under phase II trials 39. Cabozantinib, an orally bioavailable tyrosine kinase inhibitor which targeting a number of tyrosine kinases, including RET, KIT and AXL, c-Met, *etc*., has been approved for the treatment of patients with progressive, metastatic medullary thyroid carcinoma 40, 41. However, novel inhibitors are still urgently waited for anti c-Met therapy in context of breast cancer.

PARPs are a family of nuclear protein enzymes which involved in a series of cellular responses, including DNA response, gene transcription, cell proliferation and apoptosis 42. There are totally 18 family members in PARP family, and PARP-1 being the first to be characterized and most widely investigated. PARP-1 plays an important role in numerous cellular functions, such as transcriptional regulation and DNA damage repair. It is reported that PARP-1 is considered as a guardian angel against breast cancer 43. Numerous clinical studies have indicated that inhibiting PARP-1 made great effort towards personalizing treatment in breast cancer. To the best of our knowledge, there are more than 10 PARP inhibitors exerting promising treatment effect in clinical trials 44. Interestingly, previous studies have also indicated that c-Met associated with and phosphorylated PARP-1 at Tyr907, and targeting both t c-Met and PARP-1 could synergize to suppress the growth of breast cancer cells both *in vitro* and *in vivo*. Such study indicated that overexpression of c-Met may benefit from this combination therapy regardless of the cancer type 45.

In the current study, based on systems biology approaches and numerous literature researches, we manually selected c-Met and PARP-1 as the key targets in breast cancer. Subsequently, to identify inhibitors which could both targeting c-Met and PARP-1, molecular docking-based virtual screening was applied to filter 32791 candidate compounds, and MD was further calculated. Three small molecules, namely ZINC13562414, ZINC20032678, and ZINC19909930 were recognized. We synthesized these molecules and performed *in vitro* study to confirm that ZINC20032678 was the most effective small molecule which could induce two breast cancer cell lines apoptosis through inhibition of c-Met/PARP-1 kinases activities. In a word, our discovered small molecule ZINC20032678 could both target the active sites of the c-Met/PARP-1 and thus inhibiting phosphorylation and recruitment of signaling effectors, the cleaved capase-3 was activated; the expression of Bcl-2 was further inhibited while the expression of Bax was up-regulated, and eventually leading to breast cancer cell apoptosis. Therefore, ZINC20032678 could be considered as a promising and effective c-Met/PARP-1 inhibitor in future breast cancer drug discovery.

## Conclusions

In this study, a series of bioinformatics approaches were performed to identify the hub proteins, and then c-Met and PARP-1 were identified as the vital targets in breast cancer. Subsequently, *in vitro* study was carried out to confirm that the small molecule named ZINC20032678 which could both targeting c-Met/PARP-1 and induced breast cancer cell apoptosis. Our discovered targets and agents would provide new clues for the applications and modifications of future cancer drug discovery from bench to clinic.

## Figures and Tables

**Table 1:** The detailed information of hub proteins in breast cancer

Gene name	Protein name
TNKS	Poly [ADP-ribose] polymerase tankyrase-1
CCND2	G1/S-specific cyclin-D2
ITGA8	Integrin alpha-8
MET	Hepatocyte growth factor receptor
LAMA1	LAMA1 protein
IGF1	IGF1 protein
JUN	Transcription factor AP-1
ITGA1	Integrin alpha-1
RASD1	Dexamethasone-induced Ras-related protein 1
RHOU	Rho-related GTP-binding protein RhoU

**Table 2:** Molecular docking results of c-Met/PARP-1 in complex with Specs products database

ZINC number	Name	Structure
ZINC19909927	1,4-di(4-benzhydryloxy-2-butynyl)piperazine	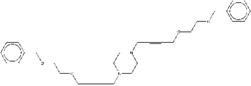
ZINC19909930	1,4-bis[4-(3-phenoxypropoxy) but-2-ynyl]piperazine	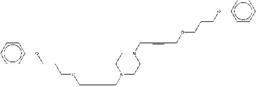
ZINC20032678	5-{1-[(12-{[1-(4-oxo-2-thioxo-1,3-thiazolidin-5-ylidene)ethyl] amino}dodecyl)amino]ethylidene}-2-thioxo-1,3-thiazolidin-4-one	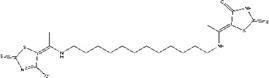
ZINC06444965	1,7-dibenzyl-8-[(2-hydroxy-1H-indol-3-yl)azo]-3-methyl-purine-2,6-dione	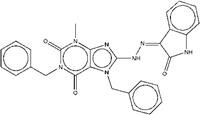
ZINC13562414	1,7-dibenzyl-8-[2-(1-{4-nitrophenyl}ethylidene)hydrazino]-3-methyl-3,7-dihydro-1H-purine-2,6-dione	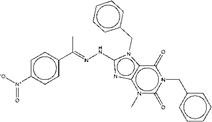
ZINC00641000	BRD-K81609421-001-01-4	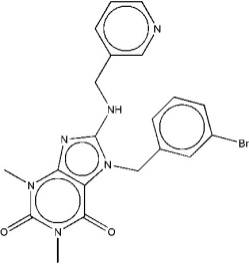
ZINC13510880	1,7-dibenzyl-8-{2-[1-(4-methoxyphenyl)ethylidene]hydrazino}-3-methyl-3,7-dihydro-1H-purine-2,6-dione	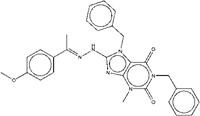
ZINC00641040	8-[(1,3-benzodioxol-5-ylmethyl)amino]-7-(3-chlorobenzyl)-1,3-dimethyl-3,7-dihydro-1H-purine-2,6-dione	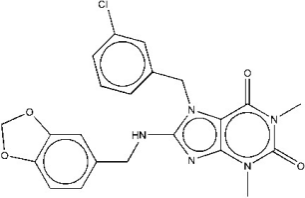
ZINC05295083	1,7-dibenzyl-3-methyl-8-[2-(1-phenylethylidene)hydrazino]-3,7-dihydro-1H-purine-2,6-dione	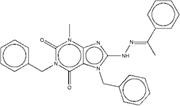
ZINC19908824	ethyl1-benzyl-5-{2-hydroxy-3-[4-(2-hydroxyethyl)-1-piperazinyl]propoxy}-2-methyl-1H-indole-3-carboxylate